# Minocycline delayed photoreceptor death in the rds mice through iNOS-dependent mechanism

**Published:** 2007-07-09

**Authors:** Li-ping Yang, Ying Li, Xiu-an Zhu, Mark O.M. Tso

**Affiliations:** 1 Peking University Eye Center, Peking University Third Hospital, Peking University, Beijing, China; 2 Wilmer Eye Institute, Johns Hopkins University School of Medicine, Baltimore, MD

## Abstract

**Purpose:**

To elucidate the role of activated microglia and nitric oxide (NO) in photoreceptor apoptosis in rds mice, and to investigate the effect of minocycline treatment on rds mice.

**Methods:**

Photoreceptor apoptosis in rds mice was detected by terminal dUTP transferase nick end labeling (TUNEL). Retinal microglial cells were identified by CD11b antibody. The mRNA expression of inducible nitric oxide synthase (iNOS) and chemokines were examined by reverse transcription polymerase chain reaction (RT-PCR) assay. The protein expression of iNOS was examined by immunohistochemistry and Western blotting analysis. The rds mice were treated intra-peritoneally from the second postnatal day (P2) with minocycline.

**Results:**

Accompanying photoreceptor degeneration in rds mice, microglia were activated and immigrated from inner retinal layer (IRL) to outer nuclear layer (ONL), and the expression of iNOS was up-regulated. Minocycline treatment reduced the iNOS expression and decreased the initial photoreceptor apoptosis, but did not provide long term ameliorative effect on the photoreceptor cell loss of rds mice.

**Conclusions:**

NO played a major role in the initial photoreceptor apoptosis in rds mice. The migration of activated microglia to the ONL contributed to the subsequent photoreceptor cell death; minocycline treatment ameliorated the photoreceptor apoptosis in rds mice, and this protective effect was partly through iNOS-suppressive mechanism.

## Introduction

Hereditary degenerative diseases of the retina lead to progressive death of rod and subsequently cone photoreceptors by apoptosis [1]. Improved understanding of the pathological process of these diseases would lead to the effective therapies to delay or ameliorate photoreceptor cell death. The rds mice is homozygous for a null mutation in the prph 2 gene, which encodes a transmembrane glyco-protein peripherin 2, which is essential for the formation and maintenance of normal photoreceptor outer segment. Consequently, the rds mice failed to develop photoreceptor outer segments and underwent progressive loss of photoreceptors by apoptosis [1,2]. In humans, over 10 different retinal phenotypes have been associated with mutations in the peripherin gene, including autosomal dominant retinitis pigmentosa (RP) and macular dystrophies (RetNet).

In the recent decade, increasing attention has been focused on the pathogenic role of microglia in retinal degenerations. Accompanying photoreceptor degenerations in Royal College of Surgeons (RCS) rat, rd mice, and light-induced retinal degeneration mice, microglia was activated and migrated from inner retinal layer (IRL) to outer nuclear layer (ONL) [3-6]. Recently, Hughes et al. observed that accompanying photoreceptor degeneration in rds mice, microglia proliferated and migrated to the subretinal space, which confirmed the intimate association between activated microglia and the degenerative process in this animal model [7,8]. However the sequential relationship between microglial activation and photoreceptor apoptosis had not been fully elucidated. The inducible nitric oxide synthase (iNOS), previously isolated from murine macrophage [9], is expressed in many cell types especially activated microglia, and induces the release of nitric oxide (NO) over long periods [10,11]. The production of NO was a major factor for tissue damage after activation of microglia. Previous studies had shown that microglial derived NO played an important role in the pathogenesis of brain ischemia [12] and experimental autoimmune uveitis [13]. Two things should be considered: (1) The possibility that iNOS might take part in the retinal degenerative process in rds mice; and (2) the co-relationship between activated microglia and iNOS expression.

Minocycline is, a semi-synthetic, long-acting tetracycline derivative that has good penetration of the blood-brain barrier. It has recently been shown to have remarkable neuroprotective properties in models of neurodegeneration [14], brain ischemia [12], Parkinson's disease [15], and multiple sclerosis [16]. Previous studies have demonstrated that minocycline treatment decreased the expression of iNOS [12] and microglial activation [17]. Recently, Hughes et al. observed that minocycline delayed photoreceptor apoptosis in rds mice [8]. The possibility that minocycline may exert its protective effect through iNOS-suppressive or microglial-suppressive mechanisms must be considered.

In the present study, we investigated the sequential events of photoreceptor apoptosis, microglial activation, and iNOS expression in rds mice. We also examined the protective effect and possible mechanisms of minocycline on the retinal degeneration process in rds mice.

## Methods

### Animals and procedures

All experiments were performed in accordance with the ARVO Statement for the Use of Animals in Ophthalmic and Vision Research. All animals were husbanded in according with the guidelines of the Association for the Assessment and Accreditation of Laboratory Animal Care. Prph2^Rd2/Rd2^ and normal control C3B mice (C3.BliA^Pdeb-rd1^) were obtained from Jackson Laboratories (Bar Harbor, ME), and were housed under specific pathogen-free conditions in the Animal Facility of Peking University Health Science Center. They were housed in an air-conditioned room with a 12:12 h light-dark cycle at a light intensity of 20 to 40 lux.

Minocycline hydrochloride (Sigma-Aldrich, St. Louis, MI) 50 mg/kg was injected intra-peritoneally into mice daily from the second postnatal day (P2) until P14, P17, P21, and P28. At least five animals and controls were used per group. A total of about one thousand mice were used.

### Tissue preparation

Animals were euthanized with an overdose of pentobarbital, and their eyes were immediately enucleated and fixed in 4% (w/v) paraformaldehyde (PFA) in PBS for 1 h. The anterior segments were removed and the posterior segments were further fixed in the same fixative for an additional period of 5 h. The tissue samples were transferred to 20% sucrose buffer overnight at 4 °C for cryoprotection and then embedded in OCT compound. Frozen sections were cut 8 μm thick through the optic nerve head and ora serrata with a cryostat, and the sections were kept in a -80 °C freezer until use.

Retinal wholemounts were prepared by bisecting the eyes at the ciliary body into anterior and posterior sections. The lens and vitreous were removed, and the retina was subsequently separated from the underling retinal pigment epithelium and choroid. Wholemount retinas were kept in a 4 °C freezer until use.

### Morphometric studies

The mouse retinas treated with or without minocycline were examined in hematoxylin and eosin-stained sections by light microscopy. For measurement of the ONL thickness, only sections passing through the optic nerve head were analyzed. Nine sections from three mice were analyzed and measurements were taken 300 μm from the optic nerve head on both sides.

### TUNEL studies

Photoreceptor apoptosis was assessed using a DeadEnd^TM^ Colorimetric TUNEL System (Promega Corporation, Madison, WI) according to manufacturers' protocol. TUNEL-positive photoreceptor cells were counted by a blind observer. Positive-stained apoptotic photoreceptors were counted in a standard length of retina (1.2 mm) centered on the optic nerve head using a graticule at 40 objective magnification following a technique as described in reference [7]. Nine sections from three mice were used for TUNEL studies.

### Immunohistochemistry studies

Experiments were carried out using appropriate positive and negative controls (no primary antibody). CD11b was used for the detection of microglia, is an antigen containing two polypeptides of 170 kDa and 95 kDa found on the surface of mouse macrophages with unknown function.

Tissue sections were fixed in chilled fresh acetone for 10 min, and were successively incubated with 0.3% H_2_O_2_ in methanol for 15 min, blocking reagents for 10 min, primary antibodies to CD11b (1:50, Serotec Ltd., Oxford, UK) or to iNOS (1:100, Santa Cruz Biotechnology, CA) at 4 °C overnight, corresponding biotinylated secondary antibodies for 45 min, horseradish peroxidase (HRP) avidin-biotin complex for 30 min and developed with DAB.

Wholemount retinas were fixed in absolute ethanol for 10 min at 4 °C, re-hydrated in 0.01 M PBS for 20 min, and made permeable by incubating in 1% Triton X-100 containing 1% bovine serum albumin for 1 h, then incubated in primary antibodies to CD11b (1:50 dilution) or to iNOS (1:100 dilution) at 4 °C overnight. Afterwards the retinas were incubated with corresponding biotinylated secondary antibodies and HRP avidin-biotin complex for 3 h at 37 °C, respectively, and developed with DAB. Retinal wholemounts were examined by confocal microscope and counted by blinded observers. Positively stained microglia in the ONL were counted in a standard area of retina using a graticule at 40 objective magnification. At least ten non-adjacent areas per eye were counted for each stain.

### Total RNA extraction and semiquantitative reverse transcription

A semiquantitative reverse transcription polymerase chain reaction (RT-PCR) assay was performed to measure the expression levels of chemokine mRNA transcripts in the whole retina. The eyes were enucleated and bisected. The retinas were peeled from the eyecup and immediately homogenized in the extraction Trizol reagent (Invitrogen Corporation, Grand Island, NY). Total RNA was isolated by chloroform extraction and isopropanol precipitation according to the manufacturer's instructions. Reverse transcription was performed with oligonucleotide primers using Superscript^TM^ reverse transcriptase (Invitrogen) according to manufacturer's protocol. Afterwards PCR was performed. The primers and annealing temperature are shown in Table 1. Each PCR product was separated on 2% agarose gel and analyzed with Quantity One 1-D Analysis Software (Bio-Rad, Richmond, CA). The amount of RT-PCR products was corrected with β-actin expression. Because of the wide variation in expression levels of these genes, we used different cycles to obtain optimum expression. The levels of expression were then compared within the time course for one particular gene mRNA, but were not compared between genes. PCR experiments were repeated at least four times.

**Table 1:** Oligonucleotides used for reverse transcriptase polymerase chain reaction.

**Target gene**	**Sequences (5'-3')**	**Location**	**Annealing temperature (°C)**	**Product size (bp)**
β-Actin	Sense: CTG GAG AAG AGC TAT GAG CTG	NT 786-1031	62	245
	Antisense: AAT CTC CTT CTG CAT CCT GTC			
iNOS	Sense: CGA CCC GTC CAC AGT ATG T	NT 402-818	57	416
	Antisense: TAC AGT TCC GAG CGT CAA AG			
MCP-1	Sense: CCC CAC TCA CCT GCT GCT ACT	NT 177-556	63	379
	Antisense: GGC ATC ACA GTC CGA GTC ACA			
MCP-3	Sense: ATA GCC GCT GCT TTC AGC A	NT 107-335	62	228
	Antisense: CTA AGT ATG CTA TAG CCT CCT CGA			
MIP-1α	Sense: CCA AAG AGA CT GGG TCC AAG	NT 307-627	63	320
	Antisense: GGG TTG AGG AAC GTG TCC TGA			
MIP-1β	Sense: CCA TGA AGC TCT GCG TGT CTG	NT 76-463	62	387
	Antisense: GGG CAG GAA ATC TGA ACG TG			
Rantes	Sense: TGC CCT CAC CAT CAT CCT CA	NT 57-366	57	309
	Antisense: AAG CGA TGA CAG GGA AGC GTA			
Eotaxin	Sense: CCT GCT GCT TTA TCA TGA CCA	NT 139-419	61	280

Primers were designed using the Primer Express Program and purchased from AuGCT Biotechnology, Beijing, P. R. China.

### Western blotting analysis

The eyes were enucleated and bisected. The retinas were peeled from the eyecup and immediately homogenized with 0.5 ml ice-cold lysis buffer [50 mM Tris-CL pH 8.0, 0.02% sodium azide, 1 μg/ml aprotinin, 1% NP-40, 100 μg/ml phenylmethylsulfonyl fluoride (PMSF)]. Insoluble material was removed by centrifugation at 120,000 rpm at 4 °C for 20 min. Final protein concentrations were determined using the BCA protein assay kit (Pierce Biotechnology, Rockford, IL) according to the manufacturer's specifications. Western blotting analysis was then performed according to a technique described in reference [18]. The antibody to iNOS, (1:1000 dilution in blocking solution) was used for immunodetection, and an anti-ERK2 polyclonal antibody was used to serve as a control to ensure that equivalent quantities of proteins were used for SDS-PAGE. Protein bands were digitally analyzed with Quantity One 1-D Analysis Software (Bio-Rad). The Western blotting experiments were repeated at least for four times.

### Statistical analysis

Data were presented as the mean±SD. An independent sample t-test was used to determine statistical significance. A p<0.01 was considered significant.

## Results

### Photoreceptor apoptosis in rds mice

**Figure 1:**
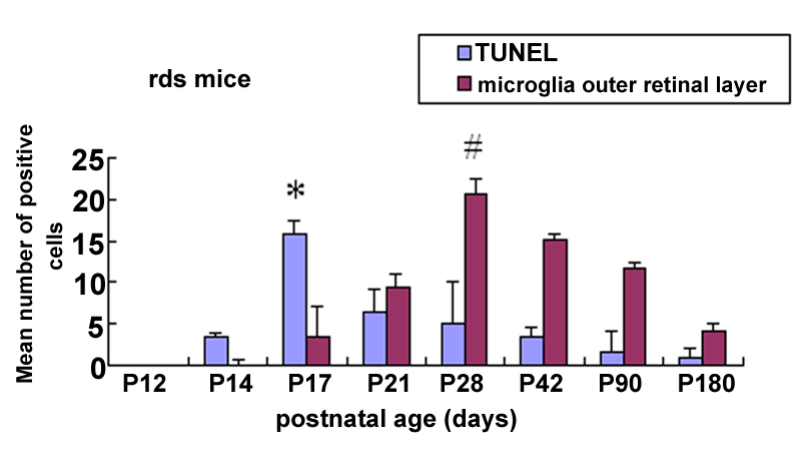
Temporal relationship between photoreceptor apoptosis and CD11b-labeled microglial cells in the outer nuclear layer of rds mouse retina. The number of TUNEL-positive photoreceptor cells peaked at P17, which was significantly higher than at P14 and P21. At P17 the activated mciroglia was first presented in the outer nuclear layer (ONL). At P28 the number of activated mciroglia in the ONL reached a peak, which was significantly higher than at P21 and P42. Data were expressed as mean±SD. A p<0.01 (*) was considered significant compared with P14 and P21 in TUNEL studies. A p<0.01 (hash mark) was considered significant compared with P21 and P42 in microglial studies.

No TUNEL-positive photoreceptor cells were observed in the ONL of the normal control C3B retina at P14. In rds mice, few TUNEL-positive cells were initially detected in the ONL at P14. At P17 the number of TUNEL-positive photoreceptor cells reached a peak. After P21 only scattered TUNEL-positive photoreceptor cells were observed. The time course of the increment and reduction of the TUNEL-positive photoreceptor cells in the ONL in rds retina are illustrated in Figure 1.

### Microglial activation in rds mice

**Figure 2:**
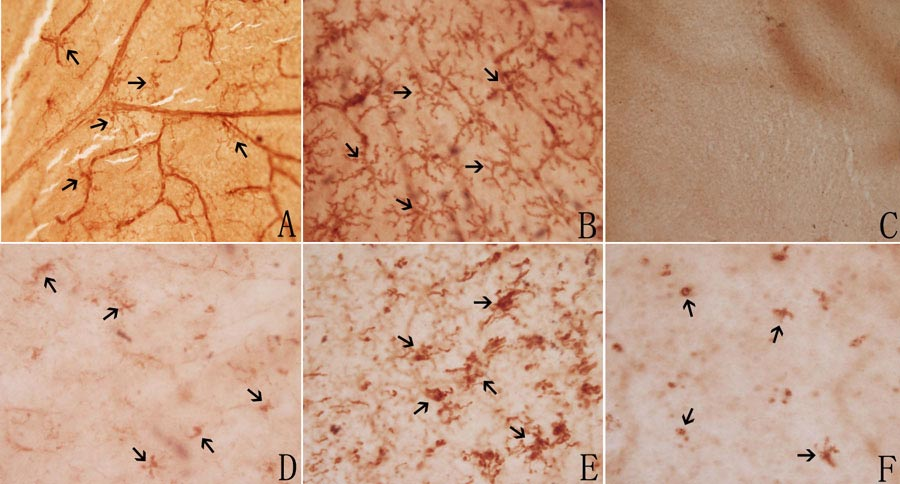
Immunohistochemical labeling of CD11b-positive microglial cells in retinal whole-mounts on control and rds mice. **A**-**C**: Normal control C3B mouse retina at P28: (**A**) In the inner retinal layer, microglia had long, thin processes, located in the peri-vascular region (arrows); (**B**) In the middle retinal layer, microglia were ramified with short, wide processes. Most cells were vessel-independent (arrows); (**C**) In the outer nuclear layer (ONL), no microglial cells were identified. **D**-**F**: Microglial cells in ONL of rds mice. The cells were ameboid with few stout processes (arrows): (**D**): At P17, microglial cells were initially present in the ONL; (**E**): By P28, the number of microglial cells in the ONL had reached their peak; (**F**): At P180, few microglial cells were scattered in the ONL. (Magnification x400).

Two subpopulations of CD11b-labeled microglial cells were identified in normal control C3B retina. One subpopulation had long-, slender processes, localized at the perivascular region in the neural fiber layer and the ganglion cell layer (Figure 2A). The other, with short wide processes, was mostly found in the retinal parenchyma, did not run along the retinal vessels and was more localized in the inner plexiform layer (Figure 2B). No microglial cells were identified in ONL in the normal control C3B retina (Figure 2C). In the rds mice before P14, the number and distribution of the microglial cells were comparable to those in the normal control C3B retina. However at P17, the microglial cells were first noted in the ONL in the rds retina (Figure 2D). At P28 the number of microglial cells in ONL reached a peak (Figure 2E). After P42 the microglial cells in the ONL were reduced in number. By P180, only a few microglial cells scattered among the ONL (Figure 2F). The microglial cells in the ONL were characteristically ameboid with few stout processes (Figure 2D-F). The temporal relationship of the increment and reduction of activated microglia in the ONL with the photoreceptor apoptosis in rds retina is illustrated in Figure 1.

### The expression of iNOS in rds retina

**Figure 3:**
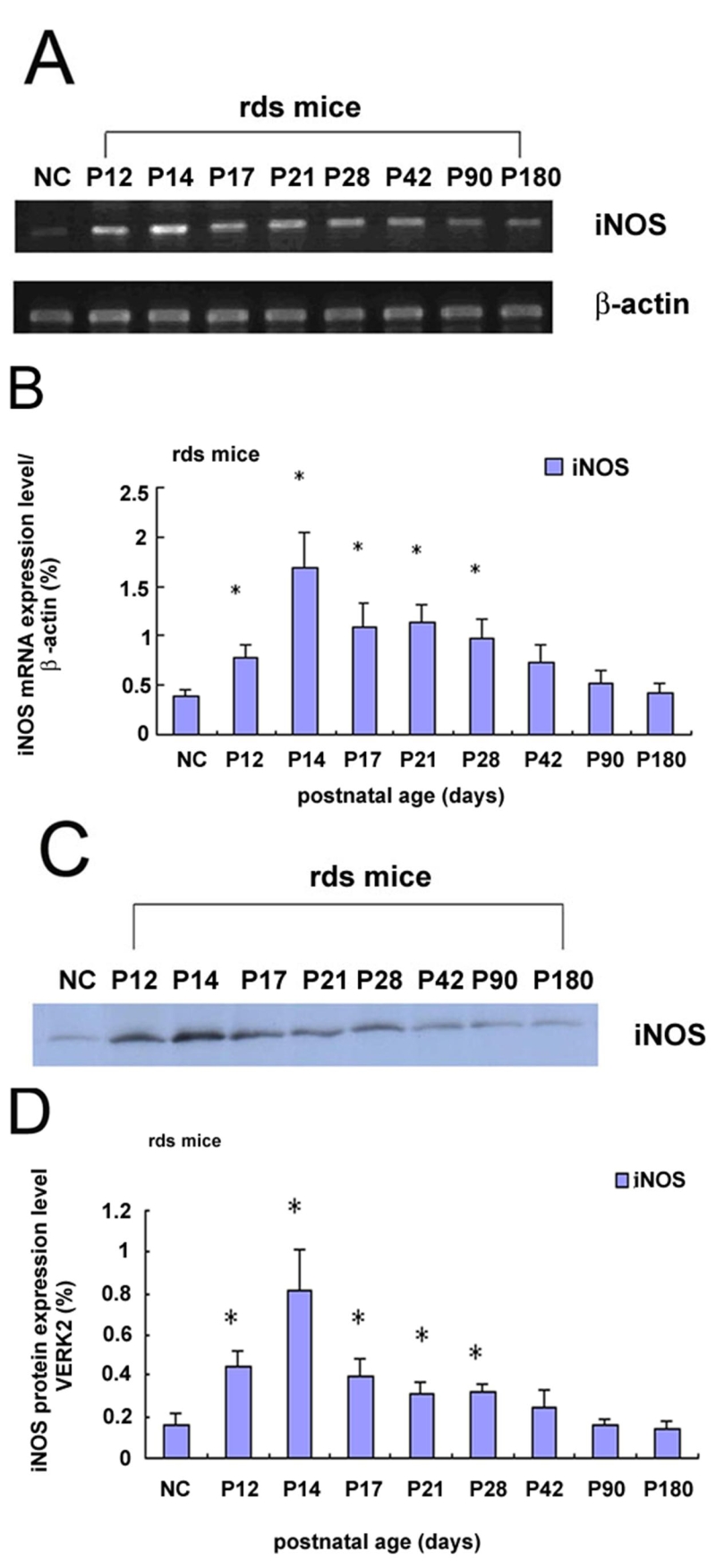
The iNOS mRNA and protein expression in control and rds mice retinas. **A**: Time course for iNOS mRNA expression in control and rds retinas for each age group. **B**: The relative levels of iNOS mRNA expression in rds mice were quantified and corrected for the level of β-actin mRNA expression. **C**: Time course for iNOS protein expression in control and rds retinas at each age group. **D**: The relative level of iNOS protein expression in rds mice were quantified and corrected for the levels of ERK2 protein expression. NC, P14 C3B mice. A p<0.01 (*) was considered significant compared with the normal control retina.

The expressions of iNOS in rds retina were determined by RT-PCR, Western blotting, and immunohistochemistry studies. A representative gel image of iNOS mRNA expression is shown in Figure 3A-B. The results showed that normal control C3B retina constitutively expressed detectable quantities of mRNA transcripts for iNOS. Accompanying photoreceptor degenerations in rds mice, the iNOS mRNA expression was up-regulated in a time dependent manner. The expression of iNOS mRNA was up-regulated from P12 and, peaked at P14. The expression slowly declined, and by P90 the expression returned to the basal level.

A representative image of iNOS protein expression was shown in Figure 3C-D. The results showed that normal control C3B retina constitutively expressed detectable quantities of iNOS protein. The expression of iNOS protein in rds retina at each age group paralleled the result of iNOS mRNA expression.

**Figure 4:**
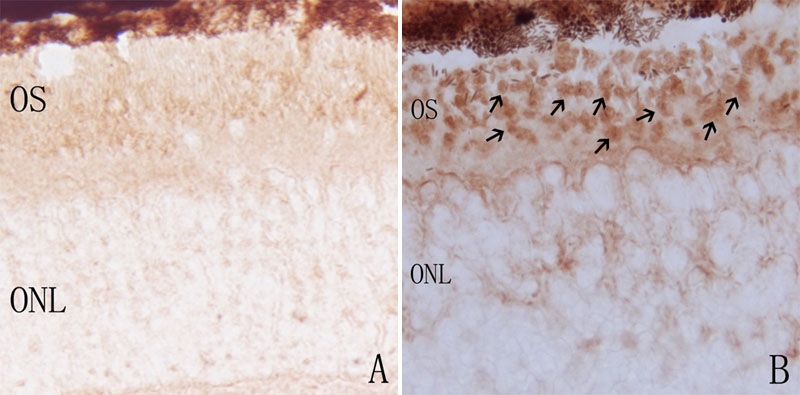
Immunohistochemical labeling of iNOS protein in control and rds mice retinas. **A**: At P14 in C3B mice, there was no iNOS-positive staining seen. **B**: At P14 in rds mice, iNOS-positive staining was mainly located at the photoreceptor outer segment (arrows). ONL represents outer nuclear layer; OS represents outer segment. (Magnification x400).

Location of iNOS protein expression was determined by immunohistochemistry study. Our study indicated that iNOS immunoreactivity was not observed in the normal control C3B retina (Figure 4A). In rds retina, the iNOS immunoreactivity was restricted to the photoreceptor outer segments (Figure 4B). Double labeling of iNOS and CD11b at the peak of microglial activation showed that iNOS was not expressed in activated microglia.

### Expression of chemokines in rds retina

**Figure 5:**
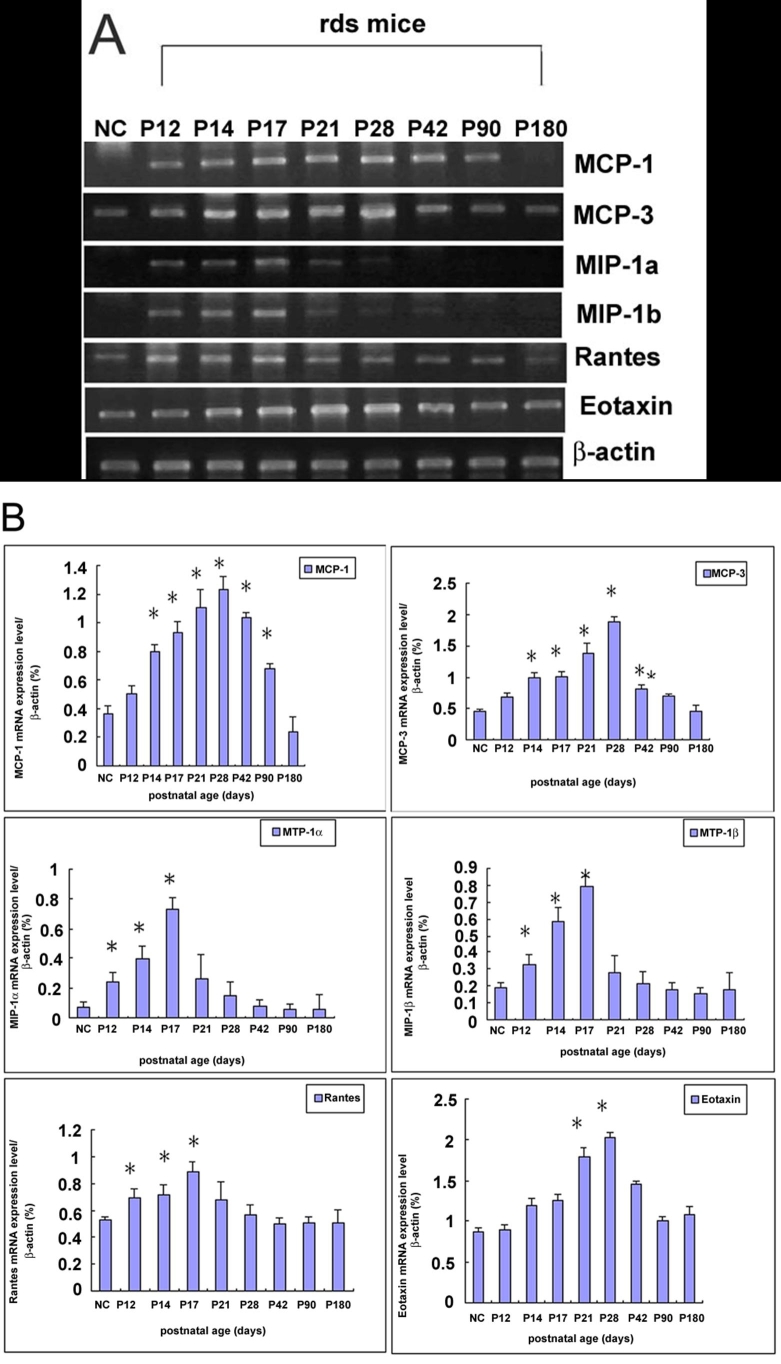
Retinal expression of chemokines in rds mice. **A**: The electrophoresis pattern of RT-PCR products for chemokines MCP-1, MCP-3, MIP-1β, MIP-1α, RANTES, and eotaxin in control and rds mice retinas. **B**: The time-course for chemokine mRNA expression in rds retina. The relative levels of mRNA were expressed as a ratio to that of β-actin. NC, P14 C3B mice. A p<0.01 (*) was considered significant compared with the normal control retina.

Relative levels of mRNA transcripts for chemokines MCP-1, MCP-3, MIP-1α, MIP-1β, RANTES and eotaxin were measured by semi-quantitative RT-PCR at various time points. A representative gel image is shown in Figure 5. The results showed that normal control C3B retina expressed low levels of mRNA transcripts for MCP-1, MCP-3, MIP-1α and MIP-1β, but expressed modest quantities of RANTES and eotaxin. In rds retina, the expression of mRNA transcripts for MCP-1, MCP-3, MIP-1α, MIP-1β, RANTES, and eotaxin was up-regulated when compared with the normal control. The expression of MIP-1α, MIP-1β and RANTES were up-regulated from P12, reached a peak at P17, and had declined to their basal levels by P28. The expressions of MCP-1, MCP-3, and eotaxin were up-regulated from P14 and, reached a peak at P28. After that time their expressions slowly declined, and by P180 had decreased to the basal level.

### Minocycline treatment reduced photoreceptor apoptosis and iNOS expression in rds mice

**Figure 6:**
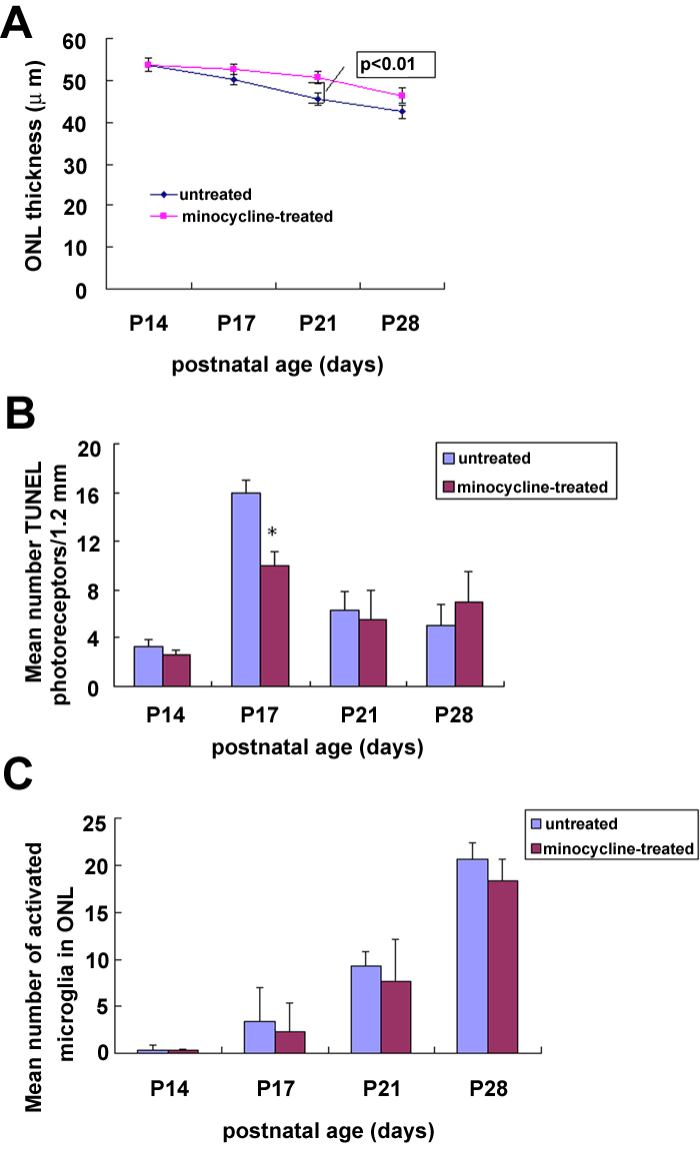
Minocycline delayed the photoreceptor apoptosis in rds mice. **A**: The retinal sections from the minocycline-treated rds eyes harbored a thicker outer nuclear layer (ONL). The difference was statistically significant at P21. **B**: The retinal sections from the minocycline-treated rds eyes displayed roughly 30% fewer TUNEL labeling at P17 than un-treated eyes. The difference was statistically significant, however the difference was statistically insignificant at P21. At P28, the number of TUNEL-positive cells in the minocycline-treated retina was more than those in the un-treated retina. The difference was statistically insignificant. **C**: Minocycline treatment had no significant effect on the number of ONL microglia in rds retina. Data were expressed as mean±SD. A p<0.01 (*) was considered significant compared with the age-paired un-treated retina.

Minocycline-treated retina harbored a thicker ONL and, was composed of more rows of photoreceptor nuclei than un-treated retina at P17, P21, and P28. The difference was statistically insignificant at P17 and P28; however, the difference was statistically significant at P21 (Figure 6A).

Photoreceptor apoptosis peaked at P17 in minocycline-treated and un-treated retinas. TUNEL-positive cells in the ONL were counted to obtain an estimate of the difference in photoreceptor apoptosis between the minocycline-treated retina and untreated controls. At P14, a few TUNEL-positive cells were detected both in minocycline-treated and un-treated retinas. The difference was statistically insignificant. At P17, the retinal sections from the minocycline-treated eyes displayed roughly 30% fewer TUNEL-positive photoreceptors than the un-treated controls. The difference was statistically significant. At P21, the retinal sections from the minocycline-treated eyes displayed fewer TUNEL-positive photoreceptors than the un-treated control eyes, but the difference was statistically insignificant. At P28, the TUNEL-positive cells in minocycline-treated eyes greater than that in the un-treated controls, and the difference was statistically insignificant. The time course of the increment and reduction of the TUNEL-positive cells in minocycline-treated and un-treated retinas is illustrated in Figure 6B.

To further examine whether minocycline treatment reduced the photoreceptor apoptosis via microglial suppressive mechanism, we investigated the effect of minocycline on the number of activated microglia in the ONL. The results showed that minocycline treatment had no significant effect on microglial activation in rds retina (Figure 6C).

**Figure 7:**
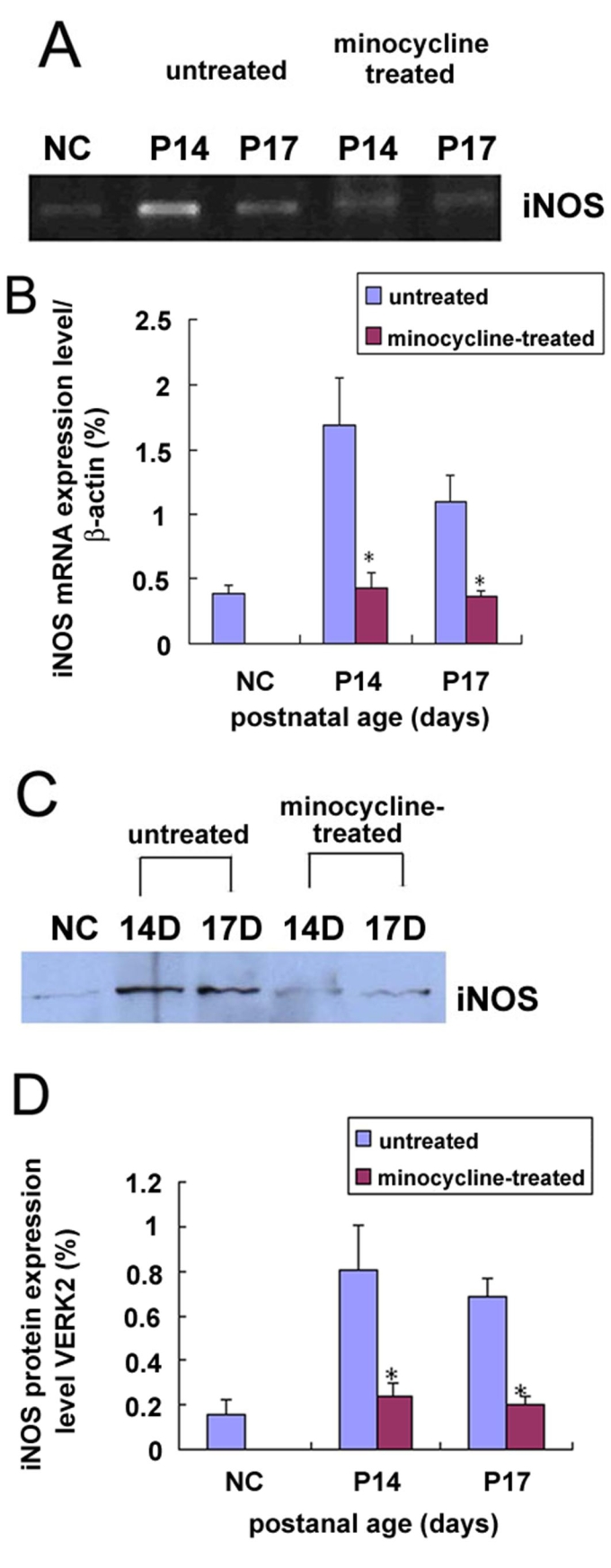
The iNOS mRNA and protein expression in minocycline-treated and un-treated rds mice. **A**, **B**: Minocycline treatment reduced the iNOS mRNA expression in rds mice at P14 and P17. The difference was statistically significant; **C**, **D**: Minocycline treatment reduced the iNOS protein expression in rds mice at P14 and P17, and the difference was statistically significant. NC, P14 C3B mice. A p<0.01 (*) was considered significant compared with the age-paired un-treated retina.

To further examine whether minocycline treatment reduced the photoreceptor apoptosis via suppression of iNOS expression, we examined the effect of minocycline on the iNOS mRNA and protein expression in rds mice. Semiquantitative RT-PCR analysis showed that administration of minocyline produced a significantly reduction in the expression of iNOS mRNA by approximately 75% and 66% at P14 and P17, respectively (Figure 7A-B). Western blotting analysis showed that administration of minocyline produced a significant reduction in the expression of iNOS protein by approximately 72% and 68% at P14 and P17, respectively (Figure 7C-D).

## Discussion

Our studies demonstrated a temporal relationship involving photoreceptor apoptosis, microglial activation, iNOS expression, and chemokine expression during the retinal degenerative process in rds mice. Photoreceptor apoptosis started at P14 and peaked at P17. Retinal microglia were activated and infiltrated ONL at P17, which was three days after the initiation of photoreceptor apoptosis, and peaked at P28, which was 11 days after the peak of photoreceptor apoptosis. The up-regulation of iNOS expression was preceded the photoreceptor apoptosis. At the peak of iNOS expression, iNOS was predominantly expressed in photoreceptor outer segment. Expression of chemokines MCP-1, MCP-3, MIP-1α, MIP-1β, RANTES and eotaxin were up-regulated during the retinal degenerative process. Minocycline treatment reduced the iNOS expression and delayed the initial photoreceptor apoptosis, but in the late stage of the disease, microglial activation and photoreceptor apoptosis persisted.

The aforedescribed sequential events suggest that NO might play an important role in the initial photoreceptor apoptosis. The microglial activation followed closely with the apoptotic process of the photoreceptors. However the initiation of photoreceptor apoptosis preceded microglial activation. So, microglia was not likely to be the initial instigator for rod apoptosis, but might influence the subsequent cone cell death in rds mice. Minocycline played its photoreceptor protective role partly through iNOS-dependent mechanism. We hypothesized that the initial gene defect in peripherin/RDS would result in the developmental disorder of photoreceptor outer segment, induce the high expression of iNOS, and secrete high levels of NO and induced the initial rod photoreceptor apoptosis. The apoptotic photoreceptors produced chemokines to activate and recruit retinal microglia to the lesion site. However the accumulation of activated microglia in the ONL not only cleared the debris, but also secreted cytotoxic factors to exaggerate subsequent cone photoreceptor death.

It is known that a low level of NO is an important mediator of homeostatic processes in the eye, such as regulation of aqueous humor dynamics [19], retinal neurotransmission [20] and photo-transduction [21]. However the production of NO at higher concentrations are toxic to the neurons [22,23]. A number of pathways could be involved in the NO-induced photoreceptor degenerative process, such as the following: (1) NO might act by eliciting a cGMP increase in the photoreceptor cells leading to excess calcium influx [21]; (2) NO could ADP-ribosilate certain photoreceptor proteins and modulate their activity [24]; and (3) NO could also act as a free radical capable of combining with oxygen derivatives, resulting in the production of the peroxynitrite anion ONOO^-^, which is highly cytotoxic [25]. In the present study the iNOS expression was up-regulated in rds mice, and its up-regulation was preceded the photoreceptor apoptosis. We propose that NO might play an important role in the initial retinal degeneration in rds mice.

Previous studies on RCS rat and rd mice suggested that activated microglia, having migrated to the photoreceptor layer in response to the debris buildup in the subretinal space or photoreceptor dysfunction, might be the instigator of photoreceptor apoptosis [3,5]. In our present study on rds mice, by closely scrutinized the temporal relationship between photoreceptor apoptosis and microglial activation, we found that the photoreceptor apoptosis was preceded the microglial activation. This was consistent with the findings by Hughes et al [8]. In addition, minocycline treatment ameliorated the initial photoreceptor apoptosis at P17, at which point activated microglia was first presented in ONL. This event, but had no effect on the subsequent photoreceptor apoptosis at P28, at which time activated microglia in ONL reached its peak. We suggest that activated microglia is innocent in the initial photoreceptor apoptosis, but might be responsible for the subsequent photoreceptor apoptosis.

Chemokines are chemotactic cytokines acting through G-protein coupled receptors. Elevated expression of chemokines have been reported in ocular inflammation [26] and light-induced retinal degeneration [27]. Recently, Zeng et al. observed that several chemokines were up-regulated in rd mice, which confirmed the intimate association of microglial activation and chemokines secretion [5]. In the present study, we observed that the expression of mRNA transcripts for MIP-1α, MIP-1β and RANTES was up-regulated from P12, peaked at P17, and declined to basal level by P28. Their expression was just correspondingly increased and declined accompanying photoreceptor apoptosis, suggested that they might be mainly secreted by apoptotic photoreceptor cells and play a major role in microglial activation and recruitment. The expression of mRNA transcripts for MCP-1, MCP-3 and eotaxin was up-regulated from P14 and peaked at P28, their expression was sustained at a relatively high level until P90, afterwards their expression was declined. The up-regulation of MCP-1, MCP-3 and eotaxin was coincided with the microglial activation, which suggested that activated microglia were the main source of these chemokines, and these chemokines might play a major role in recruiting more microglia to the lesion site.. It was noteworthy that the normal retina constitutively expressed detectable levels of RANTES and eotaxin. These two chemokines recognized the same receptor CCR3 (CC chemokine receptor 3) [28]. Previous studies demonstrated that RANTES regulated the growth and survival of first-trimester forebrain astrocyte [29]. We suggest that they were not only mediators of microglial recruitment but they might also be regulators of retinal development as suggested by Bakhiet et al. [29].

Minocycline has recently been shown to have remarkable neuroprotective properties in models of neurodegeneration [14,30]. Evidence suggests that this apparent neuroprotective property is mainly through two separate mechanisms. The first of these mechanisms was probably through direct anti-apoptotic effect. Minocycline inhibited the caspase-3 activation [8] and inhibited the release of cytochrome C from the mitochondria [31]. The second mechanism involved anti-inflammatory effects including preventing the activation and migration of microglia [30], decreasing the induction of iNOS expression [12], and reducing the cyclo-oxygenase-2 expression [32]. In the present study, we observed that minocycline treatment reduced iNOS expression and delayed the initial photoreceptor apoptosis in rds mice. Considering that NO played an important role in the initial retinal degeneration in rds mice, we suggested that minocyline treatment protect photoreceptor apoptosis partly through iNOS-dependent mechanism. However in the late stage of the disease, microglial activation and photoreceptor apoptosis persisted after minocycline treatment. The apoptotic photoreceptors produced chemokines to activate and recruit retinal microglia to the lesion site. However the accumulation of activated microglia in the ONL not only cleared the debris, but also secreted cytotoxic factors to exaggerate subsequent cone photoreceptor death. RP is initiated with the loss of night vision due to rod degeneration, and is followed by irreversible vision loss due to cone degeneration. Cone loss is responsible for the main visual handicap and its loss is indirect. Based on this study, we believe microglial suppressive factors might provide a beneficial role in these diseases.
